# The role of liquid biopsy as a catalyst for sustained progress in precision oncology – Perspective of the young committee of the international society of liquid biopsy

**DOI:** 10.1016/j.jlb.2024.100156

**Published:** 2024-05-24

**Authors:** Erick F. Saldanha, Eleonora Nicolo, Konstantinos Venetis, Diego de Miguel-Perez, Ana Ortega-Franco, Angelo Dipasquale, Mohamed A. Gouda, Surbhi Singhal, George Adigbli, Carolina Reduzzi

**Affiliations:** Division of Medical Oncology and Hematology, Princess Margaret Cancer Centre, University Health Network, University of Toronto, ON, Canada; Young Committee, International Society of Liquid Biopsy, Spain; Department of Medicine, Weill Cornell Medicine, New York, NY, 10021, USA; Young Committee, International Society of Liquid Biopsy, Spain; Division of Pathology, IEO European Institute of Oncology IRCCS, 20141, Milan, Italy; Young Committee, International Society of Liquid Biopsy, Spain; Center for Thoracic Oncology, Tisch Cancer Institute, Icahn School of Medicine at Mount Sinai, One Gustave L. Levy Place, New York, 10029, NY, USA; Translational Research Immunology Group, Nuffield Department of Surgical Sciences, University of Oxford, OX3 9DU, UK; Medical Oncology, The Christie NHS Foundation Trust, Manchester, M20 4BX, UK; Young Committee, International Society of Liquid Biopsy, Spain; Medical Oncology and Hematology Unit, IRCCS Humanitas Research Hospital, Rozzano, (Milan), Italy; Young Committee, International Society of Liquid Biopsy, Spain; Department of Investigational Cancer Therapeutics, The University of Texas MD Anderson Cancer Center, Houston, TX. 77030, USA; Young Committee, International Society of Liquid Biopsy, Spain; Division of Hematology and Oncology, Department of Medicine, University of California Davis Comprehensive Cancer Centre, Sacramento, CA, USA; Young Committee, International Society of Liquid Biopsy, Spain; Translational Research Immunology Group, Nuffield Department of Surgical Sciences, University of Oxford, OX3 9DU, UK; Young Committee, International Society of Liquid Biopsy, Spain; Department of Medicine, Weill Cornell Medicine, New York, NY, 10021, USA; Young Committee, International Society of Liquid Biopsy, Spain

## 1 Introduction

Over the past three decades, precision medicine has revolutionized oncology. At the forefront of this revolution has been the continuous expansion of liquid biopsy and its applications throughout the cancer care continuum [1,2]. Liquid biopsy refers to the evaluation of analytes in biofluids, such as circulating tumour-derived biomarkers in blood [3,4]. Compared to traditional tumour tissue analysis, liquid biopsies offer several advantages, including faster turnaround time, less invasive procedures, more comprehensive assessment of mutational profile, and feasibility for serial measurements that can better capture tumour evolution [5]. As such, liquid biopsy serves as a valuable complementary tool to tissue sampling, with a rapidly growing evidence base supporting multifaceted applications in cancer care.

In this position paper, we highlight the major advancements made in liquid biopsy and the challenges faced in its implementation within the field of oncology. We also emphasize the importance of educating and training the next generation of young clinicians and researchers to contribute to its continuous development. Finally, as members of the International Society of Liquid Biopsy (ISLB) Young Committee, we share our vision for the future of liquid biopsy in oncology.

## 2 Current applications

To date, the most established role of liquid biopsy application in the clinic is in guiding treatment selection by identifying targetable variants within a patient's tumour. Circulating tumour DNA (ctDNA) testing has been established as a successful alternative for tumour genomic profiling when tissue is unavailable [6]. The pivotal Food and Drug Administration (FDA) approval of the cobas® EGFR test v2 for screening *EGFR* (epidermal growth factor receptor) mutations from plasma cell-free DNA (cfDNA) of patients with advanced non-small cell lung cancer (NSCLC) paved the way for the approval of multiple ctDNA-based companion diagnostic (CDx) tests, such as the Guardant360 CDx and FoundationOne Liquid CDx [[7], [8], [9]]. To date, there are five FDA-approved CDx assays for treatment selection in a myriad of solid tumours [8,9]. Furthermore, compelling evidence supports the use of ctDNA for identifying mechanisms of resistance to targeted therapies in solid tumours [[10], [11], [12], [13], [14], [15]]. For instance, liquid biopsy has a high sensitivity for detecting estrogen receptor 1 (*ESR1*) mutation in metastatic estrogen receptor-positive breast cancer—a mutation primarily associated with endocrine resistance—and patients with such mutations may benefit from treatment with elacestrant [16,17]. Notably, longitudinal ctDNA can be employed to inform the treatment for metastatic colorectal cancer patients, capturing tumour heterogeneity and resistance to targeted agents. In this regard, recent evidence supports ctDNA-driven strategies to guide patient selection for the rechallenge of anti-EGFR agents [18,19].

Circulating tumour cells (CTCs) are cancer cells present in the bloodstream, which reflects the metastatic process of cancer, and are key analytes in liquid biopsies [20]. Since the seminal publication in 2004 by Cristofanilli and colleagues, demonstrating the prognostic value of CTC detection in metastatic breast cancer [21], multiple studies conducted in breast, prostate, and colorectal cancer have shown that CTC detection is associated with worse prognosis [[22], [23], [24]]. Consequently, the CellSearch platform, which enumerates CTCs in the peripheral blood of cancer patients using epithelial cell adhesion molecules (EpCAM)-based capture and fluorescent labelling [25], has received FDA approval for clinical use in patients with metastatic breast, colorectal, and prostate cancer.

Currently, only blood-based CTC and ctDNA assays are approved for routine clinical use, while other liquid biopsy assays remain investigational and should be used only in the research setting.

## 3 Challenges and future directions

While initially FDA-approved for prognosis stratification, cancer genotyping, and treatment selection in advanced cancer stages [9], liquid biopsy has the potential to play a broader role in cancer care. To this end, a range of applications are currently being evaluated, including cancer screening [26] and early cancer interception [[27], [28], [29], [30]], which could better inform treatment decisions [[31], [32], [33]]. Not surprisingly, liquid biopsies are also being leveraged to optimize drug development and the clinical trial design (NCT04457297, NCT05987241), enabling the development of personalized strategies for guiding cancer care [34].

**Fig. 1:**
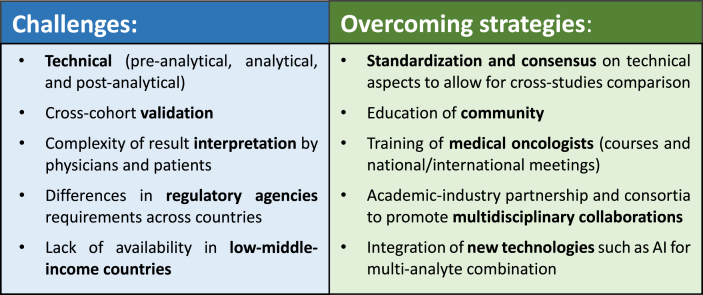
Challenges and Strategies for Widespread Implementation of Liquid Biopsy.

With reimbursement for some of these emerging indications already integrated into routine clinical practice [35], it is critical to acknowledge the existing barriers and explore ways to overcome them (Fig. 1).

Among the multiple barriers hindering the widespread incorporation of liquid biopsy in clinical practice, it is imperative to address challenges related to pre-analytical, analytical, and post-analytical phases. These include the lack of consensus regarding sample collection, collection timing, and detection thresholds, which result in a lack of clinical validation in independent cohorts and clinical trials. In addition, educating patients and clinicians on the application and interpretation of liquid biopsy tests is crucial. For instance, approved companion-based tests can encompass hundreds of genes and generate vast amounts of complex information. This can create uncertainty not only in terms of clinical application but also in the decision-making process in the clinical setting. This might also result in increased confusion/stress in patients. As such, it is vital to provide high-quality information about the tests and their application while mitigating misconceptions.

Other important considerations in liquid biopsy are the current costs and technological complexities that limit accessibility to low-middle-income countries (LMICs). We believe that accessibility to liquid biopsy technologies is critical and must be supported by academic-industrial partnerships and greater stakeholder engagement to help overcome disparities that disproportionately affect LMICs and limit access to novel tools [36].

As the role of liquid biopsy in cancer care continues to evolve, so to does the potential to interrogate cancer-related components in virtually any body fluid. Therefore, integrating analysis of various tumour-associated components in the blood using multi-omic approaches can improve the predictive performance of biomarkers. To this end, leveraging artificial intelligence (AI) and machine learning algorithms with liquid biopsy will assist in data integration and analysis, ultimately facilitating comprehensive multimodal analyses [[37], [38], [39]].

## 4 Call to action

The impact of implementing liquid biopsy in cancer care is transformative. However, to facilitate its implementation, expansion, and accessibility, a collaborative effort among patients, clinicians, providers, policymakers, and regulators is essential. The International Society of Liquid Biopsy (ISLB) has a fundamental role in this process, raising awareness of the logistical, regulatory, and educational challenges of establishing this assay in clinical care. The ISLB provides cutting-edge education and trains the next generation of scientists and clinicians to tackle the barriers to the implementation of precision medicine. To achieve this goal, the ISLB has established a comprehensive international educational program – The Certificate of Advanced Studies (CAS) in Precision Medicine. This program aims to prepare a new generation of experts in the field of liquid biopsy who can accelerate its adoption in clinical practice.

In conclusion, this position paper underscores the need for collaborative initiatives involving healthcare providers, key stakeholders, and patient advocacy groups to ensure equitable access to liquid biopsy and promote rational implementation of precision medicine strategies in oncology.

## Declaration of competing interest

The authors declare the following financial interests/personal relationships which may be considered as potential competing interests:

Carolina Reduzzi reports a relationship with International Society of Liquid Biopsy that includes: board membership. The authors in this manuscript are members of the Young Committee of the International Society of Liquid Biopsy.

If there are other authors, they declare that they have no known competing financial interests or personal relationships that could have appeared to influence the work reported in this paper.
